# Developmental white matter microstructure in autism phenotype and corresponding endophenotype during adolescence

**DOI:** 10.1038/tp.2015.23

**Published:** 2015-03-17

**Authors:** D M Lisiecka, R Holt, R Tait, M Ford, M-C Lai, L R Chura, S Baron-Cohen, M D Spencer, J Suckling

**Affiliations:** 1 Brain Mapping Unit, Department of Psychiatry, University of Cambridge, Cambridge, UK; 2 Department of Psychiatry, Behavioural and Clinical Neuroscience Institute, University of Cambridge, Cambridge, UK; 3 Autism Research Centre, Department of Psychiatry, University of Cambridge, Cambridge, UK; 4 Department of Physics, University of Cambridge, Cambridge, UK; 5 Department of Psychiatry, National Taiwan University Hospital and College of Medicine, Taipei, Taiwan; 6 Cambridge and Peterborough NHS Foundation Trust, Cambridge, UK; 7 West Suffolk Hospital NHS Trust, Bury St Edmunds, UK

## Abstract

During adolescence, white matter microstructure undergoes an important stage of development. It is hypothesized that the alterations of brain connectivity that have a key role in autism spectrum conditions (ASCs) may interact with the development of white matter microstructure. This interaction may be present beyond the phenotype of autism in siblings of individuals with ASC, who are 10 to 20 times more likely to develop certain forms of ASC. We use diffusion tensor imaging to examine how white matter microstructure measurements correlate with age in typically developing individuals, and how this correlation differs in *n*=43 adolescents with ASC and their *n*=38 siblings. Correlations observed in *n*=40 typically developing individuals match developmental changes noted in previous longitudinal studies. In comparison, individuals with ASC display weaker negative correlation between age and mean diffusivity in a broad area centred in the right superior longitudinal fasciculus. These differences may be caused either by increased heterogeneity in ASC or by temporal alterations in the group's developmental pattern. Siblings of individuals with ASC also show diminished negative correlation between age and one component of mean diffusivity—second diffusion eigenvalue—in the right superior longitudinal fasciculus. As the observed differences match for location and correlation directionality in our comparison of typically developing individuals to those with ASC and their siblings, we propose that these alterations constitute a part of the endophenotype of autism.

## Introduction

Adolescence is a period of intense increase in neural connectivity and growth of white matter (WM).^1, 2, 3, 4, 5, 6, 7^ The developmental maturation of WM microstructure is one of the most prominent intrinsic changes occurring in the adolescent brain.^3, 4, 5, 6, 7, 8, 9, 10^ Observations of both longitudinal and cross-sectional studies have consistently shown a continuous increase in fractional anisotropy (FA) and a gradual decrease in mean diffusivity (MD) in WM throughout this period.^3, 4, 5, 6, 7, 8, 10, 11, 12^ Both FA and MD represent different aspects of the diffusion tensor ellipsoid, which characterizes the average directionality of water movement observed in a voxel of a diffusion sensitive image. High FA represents high fibre coherence and directionality,^3, 13^ whereas low MD indicates fibre characteristics such as small axon calibre, high axon density or thick myelin sheath.^3, 13, 14, 15^ Therefore, high FA and low MD are associated with mature WM microstructure. The diffusion tensor ellipsoid is described by three mutually perpendicular diffusivity directions. The first (L1) is assumed to be parallel to the axonal direction of a fibre dominant in a voxel, and the second and the third (L2 and L3) are thought to be perpendicular to the axon.^16^ L2 carries additional information about the actual predominance of the water diffusion in the direction of L1 and about the possibility of crossing fibres in a voxel.^17^

Autism spectrum conditions (ASCs) arise during childhood^18^ and continue throughout adolescence.^19^ The presence of ASC during periods critical for brain growth such as early childhood and adolescence may lead to an interaction between ASC and neural development particularly in WM.^20, 21, 22, 23^ Indeed, WM disconnectivity in long-range fibres is one of the suggested pathophysiological mechanisms of ASC.^24, 25, 26^ Several studies have examined differences in WM microstructure between ASCs and typically developing children at different stages of their lives.^7, 20, 27, 28, 29, 30, 31, 32, 33^ When corrected for whole-brain multiple comparisons, younger children with ASC show increased maturation in WM microstructure in comparison with typically developing controls in the corpus callosum, cingulum, arcuate fasciculus, external and internal capsule.^29, 34^ Conversely, in later childhood and in adolescence, typically developing individuals display a more mature WM microstructure than the ASC group in the inferior and superior longitudinal fasciculi, internal and external capsule, uncinate fasciculus, cingulum, corona radiata and thalamic radiation.^27, 28, 30^ Similar trends were noted when region of interest analyses were applied.^20, 31, 32^ This indicates that the developmental pattern of WM microstructure may be affected by the presence of ASC. It also suggests that although at the beginning of puberty individuals with ASC show higher maturity in WM microstructure than typically developing controls, at the end of adolescence typically developing persons display more mature WM than the ASC group.^35^

ASC is partially heritable^36, 37, 38, 39^ and aggregates in families.^40^ Biological siblings of individuals with ASC are 10 to 20 times more likely to develop ASC than the general population.^40, 41, 42, 43^ First-degree relatives of individuals with ASC display more autistic traits, including communication and social difficulties^40, 41, 44^ in addition to rigid personality characteristics, interests and behaviour,^45^ compared with typically developing controls. This indicates that some unaffected family members of individuals with ASC inherit the associated genetic predisposition for ASC, and that they may have the broader autism endophenotype. An endophenotype is an internal characteristic present both in individuals with the condition and in their relatives, and has a genetic basis.^46^ It is an intermediate feature connecting genotype and behavioural symptoms.^46^

Variability in WM microstructure in adolescents and adults has strong genetic basis,^47, 48^ and the loci implicated in this variability (3q27 and 15q25)^48^ have also been linked to ASC susceptibility.^49^ In early and late childhood, individuals with ASC and their siblings show similar differences in WM microstructure in comparison with typically developing controls.^28^ How these differences relate to WM development during adolescence is yet to be determined. Our study aimed to test whether there is an interaction between diagnosis of ASC and age in WM microstructure in adolescents and thus determine age effects on the differences between the groups in terms of WM connectivity. We also aimed to test whether a similar interaction was observed in siblings of individuals with ASC.

## Materials and methods

### Participants

Our sample comprised 43 individuals with ASC, 38 unaffected full biological siblings of our ASC participants and 40 typically developing controls. Initially, diffusion tensor imaging (DTI) scans were obtained for 49 individuals with ASC and 40 siblings. However, six scans of participants with ASC and two scans of siblings were excluded from analysis due to motion-related artefacts and technical problems. The procedure is described in detail in the DTI analysis section. On the basis of parental report, all the siblings of individuals with ASC were their full biological siblings. The typically developing controls had no first- or second-degree relatives with ASC. The methods of reaching potential subjects and the recruitment process have been described previously.^50^

The participants with ASC were diagnosed according to the Diagnostic and Statistical Manual of Mental Disorders, fourth edition (DSM-IV), with either autistic disorder or Asperger's disorder. In addition, all participants with ASC scored above the cut-off for ‘autism' on the Autism Diagnostic Interview-Revised^51^ and for ‘autism spectrum' on the Autism Diagnostic Observation Schedule-Generic.^52^ All unaffected siblings and typically developing controls scored below the cut-off threshold (score of 15)^53^ on the Social Communication Questionnaire,^54^ a screening tool for autism.^55^ Autistic traits were quantified with the Autism Spectrum Quotient^56^ and Social Responsiveness Scale.^57^ No differences in autistic symptoms and traits were observed between typically developing controls and siblings of individuals with ASC (Table 1).

All participants were between 12 and 19 years of age and all groups were matched for age (Table 1). Both sexes were represented in all the three groups (Table 1). Typically developing individuals (20 males; 20 females) did not differ significantly in sex ratio from participants with ASC (28 males; 15 females) or from their siblings (12 males; 26 females). There was a difference in the sex ratio between the ASC and sibling groups. As individuals with ASC and their siblings were not directly compared, any difference due to their sex is of lesser importance to our conclusions. Furthermore, male siblings of individuals with ASC are at higher risk of also having an ASC diagnosis than female siblings.^58^ Intelligence quotient (IQ) was measured using the Wechsler Abbreviated Scale of Intelligence.^50, 59^ Only participants with an IQ over 70 were recruited. The mean IQ of each group was not lower than the average general population IQ.^60^ Participants with ASC had a significantly lower general IQ than the other two groups, whereas siblings and typically developing individuals did not differ in this regard (Table 1). Similar observations were seen in a previous study of cognitive abilities in siblings of children with ASC^61^ and in a study of WM maturity in adults with ASC.^62^ None of our participants had ever been medicated in relation to any psychiatric condition. The protocol was approved by the Cambridgeshire 1 Ethics Committee. All the participants and their parents have given written informed consent to participate in the study.^50^

### DTI acquisition

We used 3T MRI Magnetom TrimTrio scanner (Siemens, Erlangen, Germany) at the Medical Research Council Cognition and Brain Sciences Unit, Cambridge, UK to acquire the data. Diffusion-weighted data were obtained in 64 directions plus a single B0 (non-diffusion weighted) acquisition. Forty-eight (48) 2.5-mm slices with 20% gap (3 mm per slice in total) were acquired per direction. The voxel size was non-isotropic 1.8 × 1.8 × 2.5 mm^3^ with a field of view 230 × 230 × 144 mm^3^, TR of 6600 ms, TE of 93 ms and bandwidth of 1396 Hz per pixel.

### DTI analysis

#### Pre-processing and tensor fitting

We performed all steps of pre-processing using FMRIB Software Library (FSL).^63^ We visually inspected all the scans with FSLView. We removed from further analysis each scan with motion artefacts (‘ghosting' and severe signal loss)^64^ in over 10% of the diffusion weighted directions (seven images in total). Each slice in each diffusion direction was inspected individually and the direction was classified as compromised if three or more of its slices displayed a motion artefact. If three or more slices displayed motion artefact in the B0 image used as a baseline in calculation of the diffusion-weighted signal, the scan would have been excluded regardless of the quality of the remaining diffusion-weighted images; although in our sample, scans which displayed such signal loss in B0 also showed artefacts in more than 10% of the other images. Ultimately, scans of four participants with ASC and two siblings were removed accordingly. Two additional scans of ASC participants were removed due to low signal-to-noise ratio in the raw data, which caused them to be outliers in the number of voxels with negative MD and FA >1 (both physically implausible values). These two scans showed 8407 and 9357 voxels with negative MD as well as 3039 and 3934 voxels with FA >1 with the respective means in the remaining scans at 6397 and 2017 voxels. The low signal-to-noise ratio value in these two scans was confirmed by using B0 image and a region of interest outside the brain to calculate the signal-to-noise ratio, which was calculated as mean signal within the brain mask divided by the mean value of the noise in the region of interest outside the brain. The noise region of interest was defined manually and carefully checked to make sure it did not include skull, ghosting or other artefacts. This resulted in the ultimate sample sizes described in the Participants section.

We then corrected the data for stretches and shears produced by eddy currents and gross head motion with eddy current correction in FSL FMRIB's Diffusion Toolbox. Next, we removed all non-brain tissue from the images using Brain Extraction Tool FSL^65^ with a fractional intensity threshold of 0.2. The threshold estimating a larger brain outline was used to preserve all brain tissue and to account for variation in the participants' skull-size. Finally, we fit a diffusion tensor model to each voxel of the diffusion images with FSL FMRIB's Diffusion Toolbox DTIFit.^66^ Thus, we calculated FA, MD, L1, L2 and L3 maps for every participant.

#### Tract-based spatial statistics pre-processing

We performed all tract-based spatial statistics pre-processing in FSL.^67^ All the initial stages of tract-based spatial statistics pre-processing were performed on FA maps. First, we aligned the FA maps of all the participants to one another to identify the brain that was most representative for our sample. This approach was used to account for difference in brain proportions between our participants (children and adolescents) and an adult-derived default image used in FSL to register participants to standard space (FMRIB58_FA). The subject with the least registration warping required to align to all other subjects^68^ was selected as a target for registration to the standard space. The target was then affine-aligned to 1 × 1 × 1 mm^3^ MNI152 space. Next, the scans of other subjects underwent nonlinear transformation to the target and affine-alignment to standard space.^69, 70^ Then, the mean FA file was produced and thinned to create WM mean FA skeleton representing centres of fibres common to the entire sample.^67^ Subsequently, the 0.2 threshold was applied to produce mean FA skeleton mask representing the most prominent and consistent tracts of subjects' brains, which were later used in voxel-wise statistics.^67^ Finally, each participant's FA and non-FA (MD, L1, L2, L3) maps were projected onto the FA-derived skeleton.^67^ The resulting data were fed into intra and inter-group voxel-wise cross-subject statistics.

#### Second level analysis

First, we checked whether typically developing individuals showed patterns known to be associated with WM development by virtue of a correlation between their age and either their FA or MD, the two main WM connectivity indices with the most reliably documented developmental patterns. Then, we tested whether the neural phenotype and endophenotype of autism were characterized by alterations of typical correlations between WM measurements and age. Thus, we performed non-parametric *t*-tests comparing individuals with ASC and their siblings to typically developing participants^28^ in the correlation between age and either FA and MD and thereby tested group by age interaction for the two pairs. To further verify that the observed differences were not due to variations in IQ, we excluded six participants with the lowest IQ from the ASC group and thus equalized it among the study groups. Subsequently, we repeated comparisons between individuals with ASC and typically developing participants. Then, we compared the two pairs of groups in the correlation between age and L1, L2 and L3 to test which of the component vectors accounted most for the difference observed in the main diffusivity indices. Finally, those of the inter-group comparisons having significant results were repeated, this time controlling for sex as a confounding variable to test whether this changed the results. To perform all these comparisons, we used FSL General Linear Model tool where in one design we modelled both means and correlation with age for all the groups and FSL non-parametric randomize with 5000 permutations.^71^ Threshold-Free Cluster Enhancement with default parameters (height 2; extent 1; connectivity 26; *P*<0.05) was used to perform the whole-brain family-wise error correction for multiple comparisons in the obtained results.^72^ We defined significant results with a *P*<0.05 (threshold-free cluster enhancement corrected). JHU ICBM-DTI-81 White-Matter Labels and JHU White-Matter Tractography Atlas^73^ were used to determine in which structures the significant results were localized.

## Results

### FA and MD correlations with age in typically developing individuals

**Figure 1:**
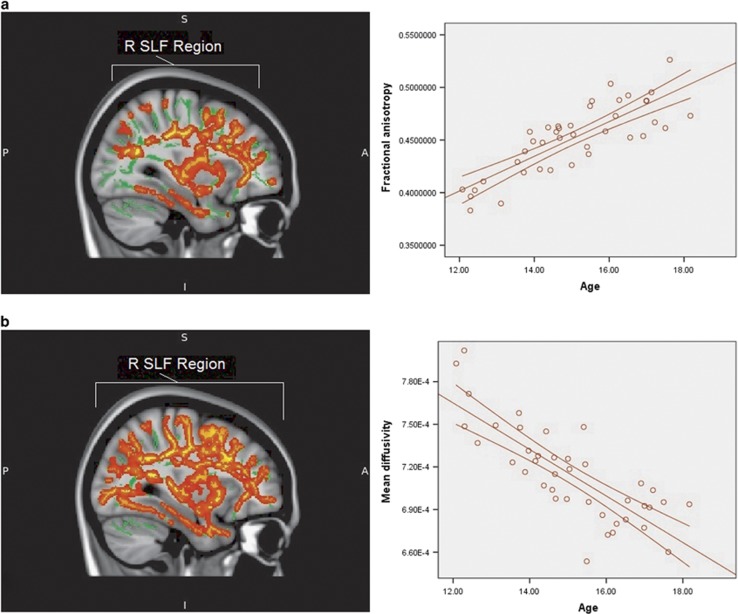
(**a**) Areas displaying positive correlation between age and fractional anisotropy values in typically developing adolescents (red area). The graph to the right presents correlation between age and fractional anisotropy values in those areas (red line). (**b**) Areas displaying negative correlation between age and mean diffusivity values in typically developing adolescents (red area). The graph to the right presents correlation between age and mean diffusivity values in those areas (red line). The results for both **a** and **b** are TFCE whole-brain corrected with a threshold at *P*<0.05. The position of the largest region is indicated. R SLF represents the right-hemispheric region centred on superior longitudinal fasciculus. Background represents the mean tract skeleton (green area) and T1-weighted MNI152 1 mm standard FSL brain (grey area). FSL, FMRIB Software Library; TFCE, threshold-free cluster enhancement.

Typically developing individuals displayed a significant positive correlation between age and FA as well as negative correlation between age and MD (Figure 1). Structures displaying correlation between FA and age involved left and right superior and inferior longitudinal fasciculus, right inferior fronto-occipital, corticospinal tract, thalamic radiation and corpus callosum (Table 2). The correlation between MD and age was observed in typically developing individuals throughout all WM and included both long- and short-range connections in both hemispheres (Table 2).

### Group differences in correlation between WM microstructure measures and age

**Figure 2:**
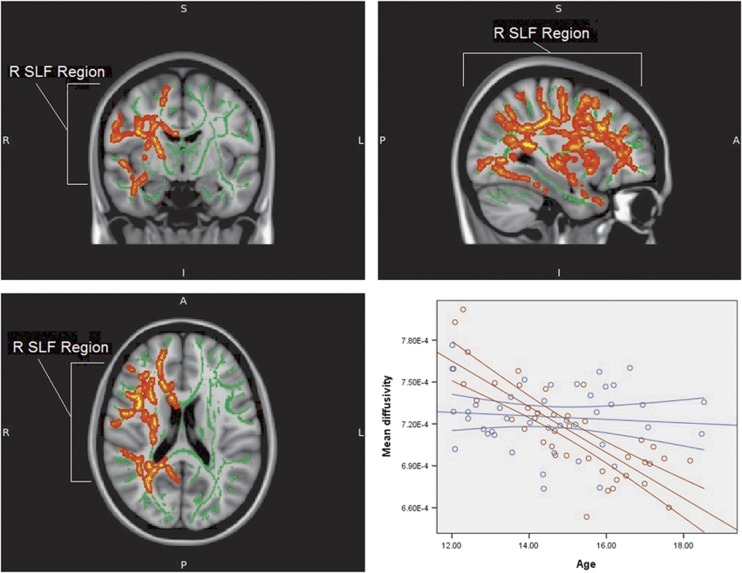
Areas displaying the difference between typically developing adolescents and individuals with ASC in correlation between age and mean diffusivity values (red area). The graph presents correlation between age and mean diffusivity values for both typically developing adolescents (red line) and individuals with ASC (blue line) in the area of significant interaction between age and diagnosis. The results are TFCE whole-brain corrected with a threshold at *P*<0.05. The position of the region is indicated. R SLF represents the right-hemispheric region centred on superior longitudinal fasciculus. Background represents the mean tract skeleton (green area) and T1-weighted MNI152 1 mm standard FSL brain (grey area). ASC, autism spectrum condition; FSL, FMRIB Software Library; TFCE, threshold-free cluster enhancement.

There was no observed interaction between age and group for age-related changes in FA. However, there was a highly significant difference between typically developing adolescents and individuals with ASC in terms of the correlation between age and MD (Figure 2). Typically developing individuals showed a far stronger negative correlation between MD and age than adolescents with ASC. The difference was observed in both short- and long-range fibres, predominantly in the right hemisphere (Table 2). After excluding the six participants with the lowest IQ scores from the ASC group, this result remained (Supplementary Material 1). The difference in age-MD correlation between the typically developing adolescents and siblings of individuals with ASC trended towards significance with the P-value of the centre of gravity of the most significant cluster at 0.059 (Supplementary Figure 2). After adding sex to the analysis as a confounding variable, the changes in the observed results were not large enough to change our conclusions (Supplementary Figure 3).

**Figure 3:**
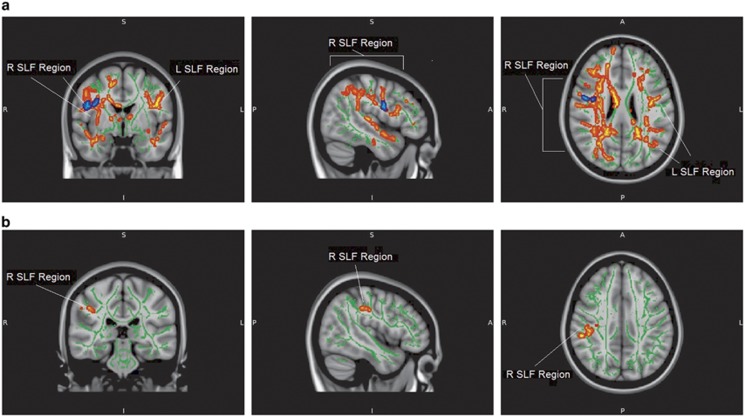
(**a**) Areas displaying the difference in correlation between age and second diffusivity direction between typically developing adolescents and individuals with ASC (red area) and between typically developing adolescents and siblings of individuals with ASC (blue area); 98.4% of the blue area is within the boundaries of the red area. (**b**) Areas displaying the difference in correlation between age and the third diffusivity direction between typically developing adolescents and individuals with ASC (red area). The results for both **a** and **b** are TFCE whole-brain corrected with a threshold at *P*<0.05. The position of the regions is indicated. R SLF represents the right-hemispheric region centred on superior longitudinal fasciculus. Background represents mean tract skeleton (green area) and T1-weighted MNI152 1 mm standard FSL brain (grey area). ASC, autism spectrum condition; FSL, FMRIB Software Library; TFCE, threshold-free cluster enhancement.

With individual tensor directions, we did not observe any difference in correlation of age and L1 between the compared groups. On the other hand, typically developing individuals showed stronger negative correlations between age and L2, as well as age and L3, in comparison with individuals with ASC with difference in L2 being observed in a broader area of the brain than the difference in L3 (Figure 3). Typically developing adolescents displayed a stronger negative correlation between L2 and age also in comparison with siblings of individuals with ASC in the right superior longitudinal fasciculus (Figure 3). Even though all the comparisons were performed for the entire brain skeleton, 98.4% of the area observed in the difference between siblings and controls spatially overlapped with the area of the difference between individuals with ASC and controls in the corresponding WM microstructure measurement (Figure 3). After adding sex to the analysis as a confounding variable, the changes in the observed results were not large enough to change our conclusions (Supplementary Figure 3).

## Discussion

For typically developing adolescents, our study found significant correlations of WM microstructure with age, corresponding to the developmental changes previously observed in longitudinal studies.^11, 12^ This suggests that these correlations are indicative of the previously observed changes in FA and in MD reported in adolescence.^3, 4, 5, 6, 7, 8, 10, 11, 12, 74, 75^ Thus, age-related development in adolescence is associated with increasing fibre coherence and directionality,^3, 13^ as well as with diminishing axon calibre, growing axon density and developing myelination,^3, 13, 14, 15^ leading to increasing consolidation and maturation of WM fibres.^4^ The change may result in a more coordinated information flow in the central nervous system^14^ and consequently in a more controlled and appropriate response to the surrounding physical and social environment of an individual.

The difference between healthy participants and individuals with ASC occurred in the correlation between MD and age and encompassed a broad area of the brain. Although at the beginning of puberty, individuals with ASC show lower MD and therefore more maturity in WM microstructure than typically developing teenagers,^29, 34^ by the end of adolescence, the trend is reversed and typically developing individuals display more mature WM than their counterparts with ASC.^27, 28, 30^ These results can be explained in two ways. First, in spite of shared core symptoms,^76^ the autistic population is heterogeneous.^77, 78^ Thus, the maturation of each individual with ASC may be idiosyncratic and possibly shifted in time for some individuals but not for the others. Therefore, when aggregated, individuals do not form a uniform line of maturation and thus large-scale longitudinal studies may be required to observe the diverse individual WM developmental trajectories. This would suggest that when genetic influences are considered, WM maturation in the ASC group could be highly impacted by loci such as 3q27 and 15q25,^48^ which contribute to individual differences in WM development and are simultaneously linked to increased susceptibility to ASC.^49^ Second, there is a possibility that WM development in the entire ASC group is shifted in time, and during adolescence it has already reached its plateau. Evidence for early brain overgrowth observed in autism^79, 80, 81, 82, 83, 84, 85^ supports this notion, suggesting that genes such as *NLGN3, NLGN4, NRXN1, CNTNAP2* and *SHANK3*—potential genetic candidates for ASC susceptibility which simultaneously affect WM development^49, 86, 87^—may contribute to the observed differences. Their selected variants predispose individuals to developing autism, potentially through shifting their WM maturation in time and thus changing the flexibility of the entire nervous system.

The differences between individuals with ASC and typically developing controls were associated with MD. This suggests that WM alterations in the phenotype of autism are related to axon calibre, density or myelination. The difference is driven by diffusivity directions perpendicular to the axon axis (L2 and L3). These measures of fibre diameter provide information about the axon membrane and its surrounding myelin sheath.^14^ The biological role of the myelin sheath is to accelerate signal transmission and to enhance fibre regeneration.^88^ During adolescence, myelination of the frontal and parietal lobes is an ongoing process.^2^ Since altered correlation between age and myelination is shown in ASC, one may speculate that signal transmission and fibre regeneration are also affected. Greater myelination observed in children with ASC may intensify information flow in some of their neural networks, reinforcing particular behaviours while limiting their variety. This may explain restricted, intense yet more advanced interests displayed by some children with autism.^89, 90^ The trend, however, reverses during adolescence and typically developing individuals are the ones with neural structures allowing for increasingly coordinated flow of information.

In our study, we observed that the largest area displaying the differences between individuals with ASC and typically developing adolescents in age-MD correlation was the right superior longitudinal fasciculus. The right superior longitudinal fasciculus is a long-range inter-lobe fibre, which underpins attentional processes in an individual.^91, 92^ According to longitudinal studies it undergoes substantial growth during adolescence.^11, 12^ Motor behaviour, visual-spatial coherence and language production, domains affected in ASC,^40^ all depend on smooth information flow in the superior longitudinal fasciculus^93^ and in each of these domains, attention has a crucial role. Impaired disengagement of attention,^94^ reduced capacity for joint attention^95, 96^ and lessened attention to social details^97^ are features central to autism. Inflexibility of attention, both situational and developmental, may prove to be a crucial feature of the mechanism leading to ASC associated with WM changes.^98^ Interestingly, withdrawing participants with the lowest IQ scores from the ASC group did not change this result, at least in our sample, providing a hint that this feature may be independent of intelligence measured with Wechsler Abbreviated Scale of Intelligence.

We also observed a difference in age-L2 correlation between typically developing adolescents and siblings of individuals with ASC. This difference was not as extensive as the one observed in phenotype and was limited to one of the perpendicular diffusivity directions which, however, carries information about predominance of the main diffusivity direction in a voxel and about the possibility of crossing fibres.^17^ The occurrence of the endophenotypic difference in L2 suggested that individuals with ASC and their siblings may display less crossing fibres in the right superior longitudinal fasciculus than typically developing teenagers at the beginning of adolescence but this trend reversed at the end of this period. What is important the endophenotypic difference followed the same pattern, with typically developing individuals displaying stronger negative correlation between L2 and age than siblings of individuals with ASC in the right superior longitudinal fasciculus, at mostly the same region as that found in the ASC-control comparison. A similar relation between individuals with ASC and their siblings, where the latter displayed a milder form of the changes characteristic for the former, was observed in a younger cohort by Barnea-Goraly *et al.*^28^ As the differences noted in first-degree relatives are a more subtle variant of those observed in the autism phenotype, it is possible that alterations in WM maturation aggregate with increasing presence of genetic mutations associated with ASC. However, autistic symptoms appear when the level of WM alterations surpasses a certain threshold.^46^ Therefore, although these features form a continuum, the diagnosis can be disjunctive. The detected difference is especially striking as siblings of individuals with ASC did not differ in autism symptoms from typically developing adolescents. It suggests that these alterations can be more fundamental than observed symptoms.

### Limitations

In our study, we observed some differences between the diagnostic groups in the distribution of sex and IQ. This poses a question as to whether such features of the samples might be responsible for the differences noted in WM. Differences of distribution in IQ have been observed in previous studies of ASC, and are difficult to control through statistical modelling.^61, 62^ This is not to say that the ASC group displays lower general intelligence than the typically developing population, but rather that individuals of this group may have specific difficulties related to Wechsler Abbreviated Scale of measured intelligence.^99^ However, after excluding the participants with lowest IQ scores from the ASC group, and thus diminishing the difference in IQ between the two groups to a level of non-significance, we still observed the difference between the groups in WM. This suggests that the observed differences in MD go beyond the differences in IQ. Dissimilarity of sex ratio is of lesser importance for our conclusions as we did not investigate the difference between individuals with ASC and their siblings directly, which were the only two groups to differ significantly in gender distribution. Furthermore, after adding sex to the analysis as a confounding variable, the changes in the observed results were not large enough to change our conclusions.

Our study is additionally limited by the fact that it uses a cross-sectional design to observe changes associated with age. However, our observations in typically developing individuals concord with the results of previous longitudinal studies in this age group.^11, 12^ Furthermore, entry of our participants to the examined groups were statistically independent in age, thus preventing any bias linked with non-random entry of the participants into the sample.^100^ We cannot entirely guarantee that age-trajectories of all individuals in the ASC group or in the siblings group were parallel, and therefore we cannot ascertain whether weaker correlations between diffusivity measures and age in these groups indicate changes in the development trajectory of the entire group or increased developmental variability within each group. For this reason we offer two possible explanations of the observed differences in the Discussion section above.^100^

## Conclusion

We conclude that the correlation between age and WM microstructure measurements differs between individuals with and without ASC. The difference in its milder form also appears in siblings of individuals with ASC, compared with the typically developing population, suggesting that WM development during adolescence may be a potential endophenotype of autism and may be associated with altered number of crossing fibres. The difference is observed in areas that have been associated with attention and cognitive flexibility in the typically developing population. This suggests that attention flexibility and its association with WM development may be an interesting topic for future studies of the ASC endophenotype. Our findings emphasize the importance of WM development in pathophysiology of ASC and in integrating its genes-to-symptoms mechanisms. They also signal that a potential treatment for ASC may be extended to the period of adolescence.

## Figures and Tables

**Table 1:** Demographic and clinical characteristics of the study groups

	*ASC* (N=*43)*	*Siblings* (N=*38)*	*Controls* (N=*40)*	*Group differences*
Age (years)	14.52±1.74	14.99±2.13	15.06±1.63	Not significant
Gender (M/F)	28/15	12/26	20/20	No significant difference between controls and the other two groups Significant difference between ASC and siblings; *P*=0.002
Wechsler Abbreviated Scale of Intelligence	104.65±14.57	113.63±9.95	112.38±11.12	No significant difference between siblings and controls Significant differences between: ASC < Siblings; *P*=0.003 ASC < Controls; *P*=0.012
Social Communication Questionnaire	25.03±5.5	1.6±2.19	2.2±2.33	No significant difference between siblings and controls Significant differences between: ASC > Siblings; *P*<0.001 ASC > Controls; *P*<0.001
Autism Spectrum Quotient	38.59±6.72	8.61±5.63	9.1±5.59	
Social Responsiveness Scale	112.85±31.13	17.22±12.34	15.43±11.4	

Abbreviations: ASC, autism spectrum condition; F, female; M, male. Interval measurements are presented as a group mean with s.d. The significance of differences between the groups means in every measurement was calculated with *t*-tests for each pair of the groups.

**Table 2:** Areas displaying significant correlations between white matter microstructure measurements and age

*Tracts*	*Cluster size (*N *voxels)*	T *value*	P*-value (TFCE corrected)*	*Coordinates of the peak in aligned anatomical space*		
				*X*	*Y*	*Z*
*Positive correlation between fractional anisotropy and age in typically developing individuals*						
Superior longitudinal fasciculus R External capsule R Corpus callosum (forceps minor, body, splenium, tapetum, forceps major) Inferior fronto-occipital fasciculus R Inferior longitudinal fasciculus R Corona radiata (anterior, superior and posterior part) R Internal capsule (anterior and posterior limb, retrolenticular part) R Thalamic radiation (anterior and posterior part) R Fornix R	15 032	2.66	0.021	42	−13	−14
Inferior longitudinal fasciculus L Corticospinal tract L Thalamic radiation (anterior and posterior part) L Medial lemniscus L Inferior fronto-occipital fasciculus L Superior longitudinal fasciculus L Cingulum (cingulate gyrus and hippocampus) L Posterior corona radiata L	2033	3.63	0.026	−24	−71	26
Corticospinal tract R Anterior thalamic radiation R	1628	3.22	0.039	9	−29	−19
Cingulum in hippocampus R Inferior longitudinal fasciculus R	1102	3.84	0.034	30	−20	−28
Uncinate fasciculus R Inferior fronto-occipital fasciculus R	241	3.69	0.042	28	14	−9
Cerebellum (peduncles and pons)	98	3.94	0.046	11	−75	−33
Anterior thalamic radiation R Medial lemniscus R	80	3.46	0.048	8	−20	−8
Inferior longitudinal fasciculus L	72	3.76	0.046	−34	−57	−12
Forceps major Inferior fronto-occipital fasciculus R	66	2.89	0.047	24	−87	23
Inferior fronto-occipital fasciculus R	21	2.79	0.049	28	48	−6
Middle cerebellar peduncle	12	3.37	0.049	14	−29	−37
Inferior longitudinal fasciculus L	4	3.53	0.049	−34	−48	−14
						
*Negative correlations between mean diffusivity and age in typically developing individuals*						
Corpus callosum (forceps minor, genu, body, splenium, tapetum, forceps major) Superior longitudinal fasciculus R+L Cerebellum (peduncles and pons) Corona radiata (anterior, superior and posterior part) R+L Inferior fronto-occipital fasciculus R+L Internal capsule (anterior and posterior limb, retrolenticular part) L+R Inferior longitudinal fasciculus R+L Corticospinal tract R+L Thalamic radiation (anterior and posterior part) R+L External capsule R+L Cingulum (cingulate gyrus and hippocampus) R+L Uncinate fasciculus L+R Medial lemniscus R+L Fornix L+R	79 764	2.12	<0.001	16	−79	22
						
*Negative correlation between mean diffusivity and age: ASC < typically developing individuals*						
Superior longitudinal fasciculus R Corona radiata (anterior, superior and posterior part) R Corpus callosum (forceps minor, genu, body, splenium, tapetum, forceps major) Inferior fronto-occipital fasciculus R External capsule R Inferior longitudinal fasciculus R Thalamic radiation (anterior and posterior part) R Internal capsule (anterior and posterior limb, retrolenticular part) R Corticospinal tract R Cingulum (cingulate gyrus and hippocampus) R Uncinate fasciculus R Fornix R	20 963	3.38	0.011	20	−74	37
						
*Negative correlation between second diffusivity direction and age: ASC < typically developing individuals*						
Superior longitudinal fasciculus R Corona radiata (anterior, superior and posterior part) R Corpus callosum (forceps minor, genu, body, splenium, tapetum, forceps major) Corticospinal tract R Thalamic radiation (anterior and posterior part) R Inferior fronto-occipital fasciculus R Internal capsule (anterior and posterior limb, retrolenticular part) R External capsule R Inferior longitudinal fasciculus R Cingulum (cingulate gyrus and hippocampus) R Uncinate fasciculus R Fornix R	14 391	2.82	0.017	50	0	18
Corpus callosum (splenium and forceps major)	4924	3.68	0.031	−20	−49	22
Anterior thalamic radiation L	4489	3.24	0.037	−6	−4	8
Forceps minor Anterior thalamic radiation R	3835	3.98	0.029	20	37	19
Uncinate fasciculus L Inferior fronto-occipital fasciculus L Anterior thalamic radiation L	1622	3.24	0.038	−39	38	6
Inferior longitudinal fasciculus L Uncinate fasciculus L Superior longitudinal fasciculus L	807	3.63	0.04	−44	3	−21
Superior longitudinal fasciculus L	770	3.87	0.047	−39	6	42
Inferior longitudinal fasciculus L Inferior fronto-occipital fasciculus L Superior longitudinal fasciculus L	406	2.45	0.047	−42	−17	−17
Forceps minor Cingulate gyrus Anterior corona radiata L	275	2.57	0.047	−17	33	14
Corticospinal tract L	249	3.98	0.043	−19	−31	48
Inferior longitudinal fasciculus L	232	3.03	0.048	−34	−54	−13
Middle cerebellar peduncle	128	3.18	0.048	24	−65	−37
Inferior fronto-occipital fasciculus L Inferior longitudinal fasciculus L Forceps major	94	2.3	0.048	−26	−72	0
Inferior longitudinal fasciculus L Uncinate fasciculus L	91	2.68	0.049	−40	−3	−24
Corticospinal tract L	81	2.88	0.048	−9	−29	60
Uncinate fasciculus L	61	3.08	0.049	−26	22	−17

*Negative correlation between second diffusivity direction and age: ASC < siblings*						
Superior longitudinal fasciculus R	77	2.68	0.041	47	−3	24
Superior longitudinal fasciculus R	47	2.84	0.047	34	−2	29
						
*Negative correlation between third diffusivity direction and age: ASC < typically developing individuals*						
Superior longitudinal fasciculus R	68	4	0.046	53	−33	36
Superior longitudinal fasciculus R	67	3.85	0.047	42	−28	37

Abbreviations: ASC, autism spectrum condition; L, left; R, right; TFCE, threshold-free cluster enhancement.
