# Supplementary figures and images for: Developmental white matter microstructure in autism phenotype and corresponding endophenotype during adolescence
Source: Transl Psychiatry. 2015 Mar 17;5(3):e529–. doi: 10.1038/tp.2015.23 (PMC4354353; doi:10.1038/tp.2015.23)

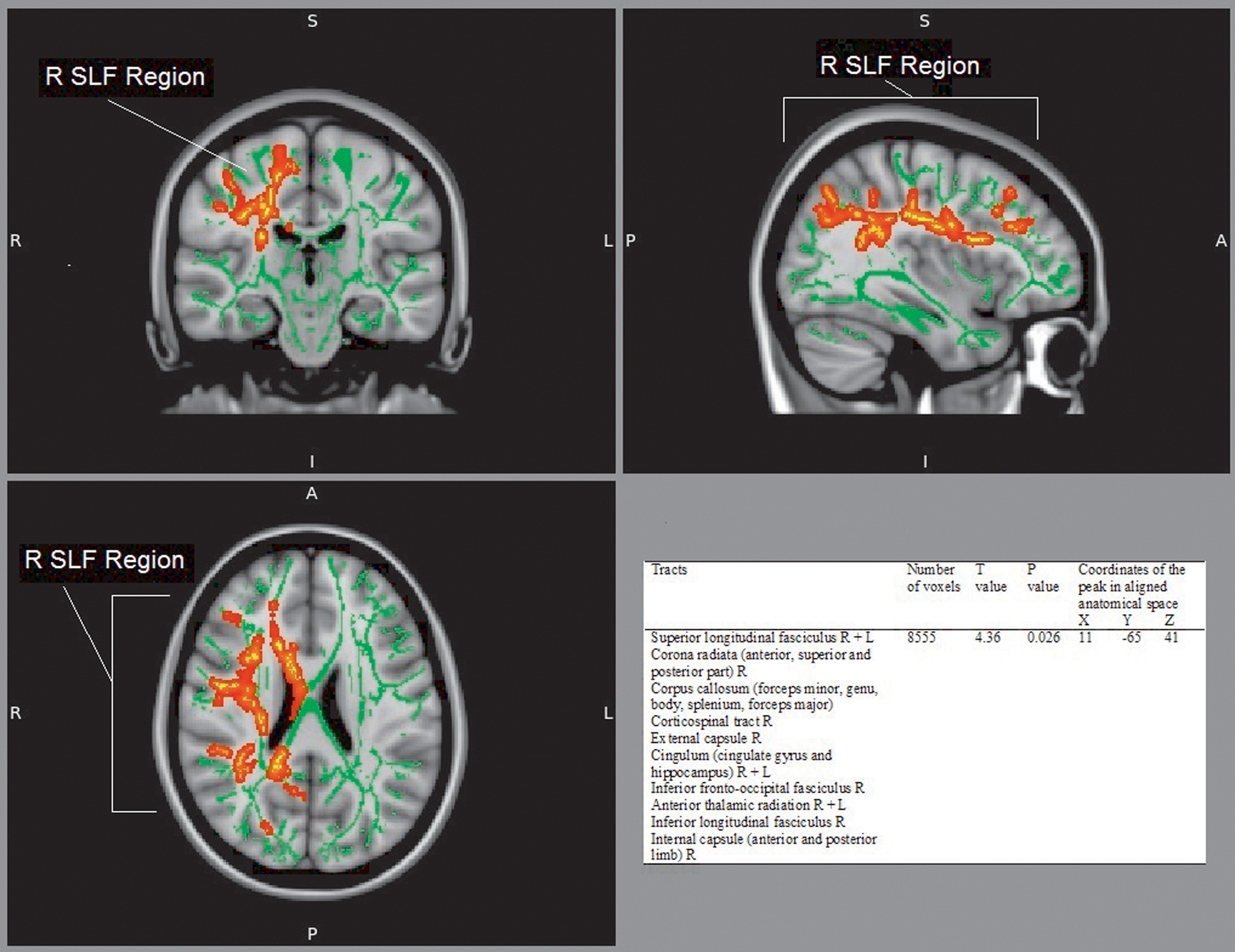

Supplement: Supplementary Figure 1 [file tp201523x1.tif]

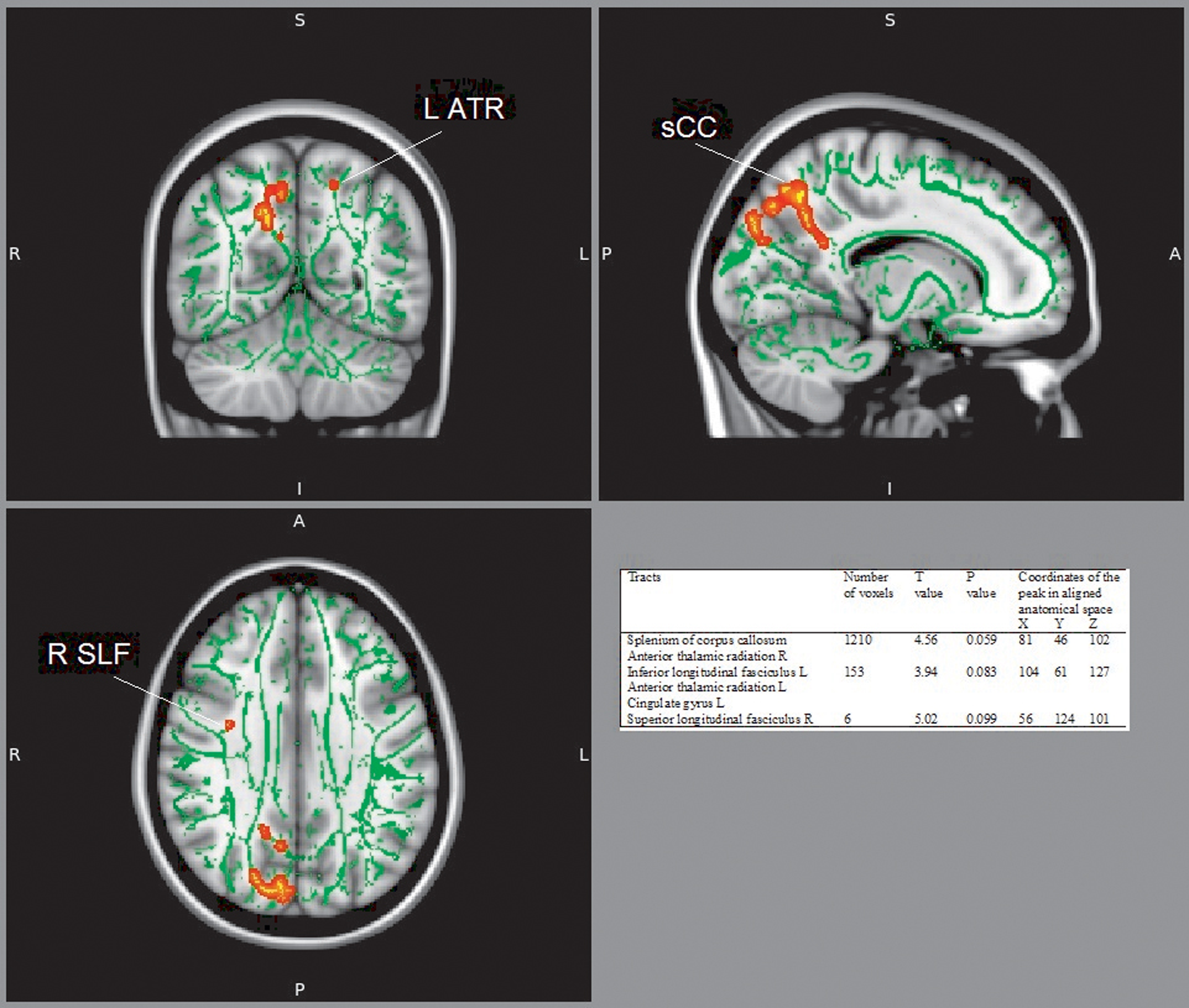

Supplement: Supplementary Figure 2 [file tp201523x2.tif]

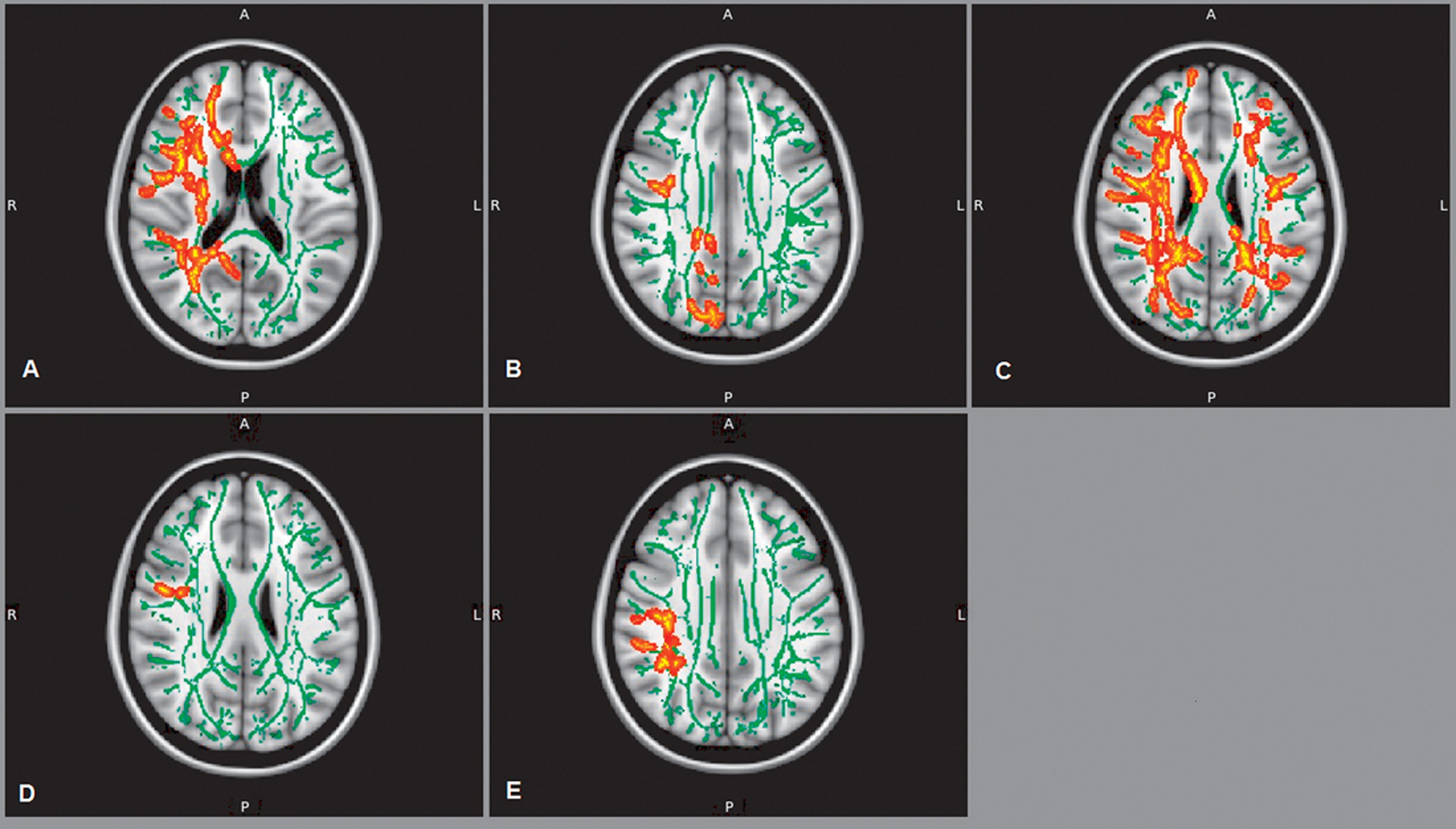

Supplement: Supplementary Figure 3 [file tp201523x3.tif]
