# Supplementary material for: Developmental white matter microstructure in autism phenotype and corresponding endophenotype during adolescence
Source: Transl Psychiatry. 2015 Mar 17;5(3):e529–. doi: 10.1038/tp.2015.23 (PMC4354353; doi:10.1038/tp.2015.23)
Supplement: Supplementary Figure Legends [file tp201523x4.doc]

Supplementary figure 1:

Areas displaying the difference between typically developing adolescents and individuals with ASC in the correlation between age and mean diffusivity after six participants with lowest IQ scores were excluded from ASC group (red area). The graph presents correlation between age and mean diffusivity for both typically developing adolescents (red line) and individuals with ASC (blue line) in the area of significant interaction between age and diagnosis. The results are TFCE whole-brain corrected with a threshold at p<0.05. The position of the region is indicated. R SLF Region: the right-hemispheric region centred on superior longitudinal fasciculus. Background: mean tract skeleton (green area) and T1-weighted MNI152 1mm standard FSL brain (grey area).

Supplementary figure 2:

Areas displaying the difference between typically developing adolescents and siblings of individuals with ASC in correlation between age and mean diffusivity (red area). The results are TFCE whole-brain corrected with a threshold at p<0.1. The positions of the regions are indicated. R SLF: the right superior longitudinal fasciculus; L ATR: left anterior thalamic radiation; sCC: splenium of corpus callosum. Background: mean tract skeleton (green area) and T1-weighted MNI152 1mm standard FSL brain (grey area).

Supplementary figure 3:

A) Areas displaying positive correlation between age and fractional anisotropy in typically developing adolescents with sex as a confounding variable (red area). The results are TFCE whole-brain corrected with a threshold at p<0.05. B) Areas displaying the difference between typically developing adolescents and siblings of individuals with ASC in correlation between age and mean diffusivity (red area). The results are TFCE whole-brain corrected with a threshold at p<0.1. C) Areas displaying the difference in correlation between age and second diffusivity direction between typically developing adolescents and individuals with ASC (red area). The results are TFCE whole-brain corrected with a threshold at p<0.05. D) Areas displaying the difference in correlation between age and second diffusivity direction between typically developing adolescents and siblings of individuals with ASC (red area). The results are TFCE whole-brain corrected with a threshold at p<0.05. E) Areas displaying the difference in correlation between age and the third diffusivity direction between typically developing adolescents and individuals with ASC (red area). The results are TFCE whole-brain corrected with a threshold at p<0.05. Background: mean tract skeleton (green area) and T1-weighted MNI152 1mm standard FSL brain (grey area).
