# Supplementary material for: Developmental white matter microstructure in autism phenotype and corresponding endophenotype during adolescence
Source: Transl Psychiatry. 2015 Mar 17;5(3):e529–. doi: 10.1038/tp.2015.23 (PMC4354353; doi:10.1038/tp.2015.23)
Supplement: Supplementary Information [file tp201523x5.doc]

# Supplementary material 1

We repeated the comparison of correlation between age and mean diffusivity between individual with ASC and typically developing adolescents after excluding the six participants with lowest IQ scores from the ASC group. This was the lowest possible number of participants that resulted in matching the ASC group to the other two groups in the study in relation to WASI-measured IQ. The mean IQ in the ASC group was now 107.89 (SD=13.01) and was not significantly different from the one of their siblings (p=0.079) and of typically developing individuals (p=0.201). The excluded participants were three males and three females and the difference in gender ratio between individuals with ASC and typically developing adolescents was not significant.

In the age-related decrease in mean diffusivity, we observed a difference similar to the one noted with the full set of the participants (Supplementary figure 1). Hence, we concluded that the difference observed in our main analysis was not attributable to any observed difference in intelligence between the groups.
